# Immunometabolic crosstalk between dysfunctional adipose tissue and the brain: implications for postoperative cognitive dysfunction in obesity

**DOI:** 10.3389/fimmu.2026.1965479

**Published:** 2026-10-12

**Authors:** Ya-Jiao Hu, Juan Ni, Jin-Xi Zhang

**Affiliations:** 1 Department of Anesthesiology, West China Second University Hospital, Sichuan University, Chengdu, Sichuan, China; 2 Key Laboratory of Birth Defects and Related Diseases of Women and Children, Ministry of Education, Sichuan University, Chengdu, Sichuan, China

**Keywords:** adipose tissue immune remodeling, blood–brain barrier, immunometabolism, neuroinflammation, obesity, peripheral immune activation, postoperative cognitive dysfunction

## Abstract

Postoperative cognitive dysfunction (POCD) is an important complication after surgery, particularly in patients with pre-existing metabolic and inflammatory vulnerability. Obesity may increase susceptibility to postoperative cognitive decline, but its independent association with POCD and the underlying mechanisms remain uncertain. This review critically examines a proposed adipose–immune–brain pathway linking obesity-associated adipose dysfunction to perioperative neurocognitive outcomes. In obesity, adipocyte hypertrophy, hypoxia, fibrosis, macrophage accumulation, T-cell imbalance, and activation of inflammasome and innate immune signaling pathways promote chronic low-grade inflammation and altered endocrine function. The resulting changes in cytokines, adipokines, free fatty acids, lipid mediators, insulin sensitivity, and glucose and lipid metabolism may establish a systemically primed immunometabolic state. Surgical trauma may then act as a second inflammatory hit, amplifying peripheral immune activation and promoting blood–brain barrier dysfunction, glial activation, oxidative stress, and impaired neuronal function. However, direct clinical evidence linking adipose tissue inflammation to POCD remains limited; current support is derived largely from preclinical studies, indirect biomarker associations, and heterogeneous clinical studies that sometimes combine postoperative delirium with longer-term cognitive outcomes. Accordingly, the adipose–immune–brain axis should be regarded as a biologically plausible framework rather than an established causal pathway. Better phenotyping of visceral adiposity and metabolic dysfunction, standardized neurocognitive outcomes, and integrated assessment of adipose, circulating, and blood–brain barrier biomarkers are needed. Potential interventions include weight and metabolic optimization, incretin-based therapies, modulation of inflammatory and pro-resolving pathways, and perioperative physiological management, although their efficacy for preventing POCD remains to be established.

## 1 Introduction

Postoperative cognitive dysfunction (POCD) refers to a persistent decline in cognitive function following surgery, and its clinical significance has been increasingly recognized in recent years (1–3). With an aging global population, the reported incidence of POCD in individuals over 55 years ranges between 5% and 56%, and the incidence at 3 months post-surgery is approximately 10%–17% (4). Although POCD has been extensively studied after cardiac surgery, The International Study of Post-Operative Cognitive Dysfunction 1 (ISPOCD1) found that among patients 60 years of age or older undergoing noncardiac major surgery, the incidence of POCD is 25.8% at one week and 9.9% at three months after surgery (4, 5). The risk of POCD is multifactorial including age, surgical trauma, anesthesia, hypotension, metabolic disorders, and neurological disorders (4, 6–10). Although the effect of surgery type remains controversial, some studies suggest that major cardiac surgery is associated with higher POCD rates; additional contributors may include longer surgery duration, postoperative complications, heightened stress responses, and prolonged hospitalization (11, 12).

Obesity, a globally prevalent chronic disease, has been identified as a risk factor for cognitive impairment and may be associated with POCD, although the specific link between obesity and POCD is not yet fully elucidated (13–16). Available clinical evidence suggests that postoperative cognitive outcomes are influenced by multiple interrelated systemic factors, including metabolic burden, oxidative stress, malnutrition, and frailty (17–19). Because several of these factors may coexist with obesity, they may contribute to or confound the observed association between obesity and postoperative cognitive vulnerability. However, the current evidence does not establish obesity itself as an independent risk factor for POCD. This review summarizes current evidence on how obesity-induced adipose immune remodeling and immunometabolic dysfunction promote systemic immune activation and neuroinflammation, potentially increasing susceptibility to POCD. Understanding these pathways is critical for identifying high-risk patients, developing early biomarkers, and designing targeted therapeutic and preventive strategies.

This review therefore examines the proposed sequence from obesity-induced adipose immune remodeling to systemic immunometabolic dysfunction, perioperative inflammatory amplification, blood–brain barrier (BBB) disruption, and postoperative cognitive vulnerability (**Figure 1**).

**Figure 1:**
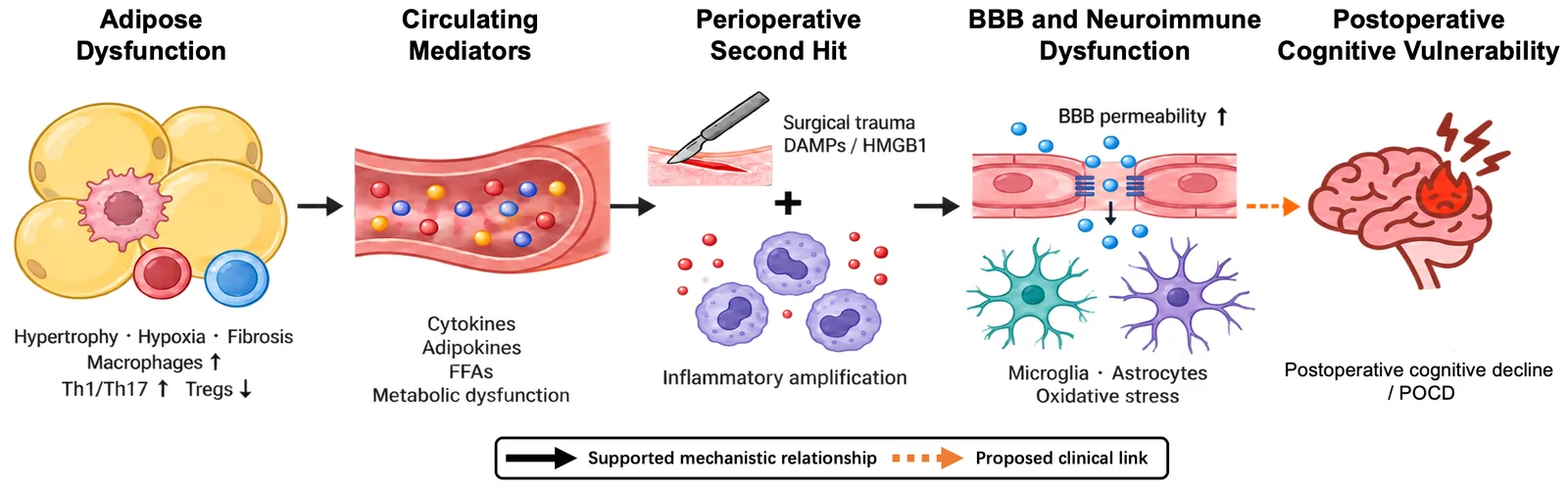
Conceptual second-hit model linking obesity-associated adipose dysfunction to postoperative cognitive vulnerability. Obesity-associated adipose hypertrophy, hypoxia, fibrosis, macrophage accumulation, T-cell imbalance, and reduced regulatory T-cell activity may establish a chronically primed immunometabolic state. Circulating cytokines, adipokines, free fatty acids, and metabolic abnormalities transmit these peripheral signals. Surgical trauma and the release of damage-associated molecular patterns, including high-mobility group box 1, may subsequently act as a second inflammatory hit and amplify peripheral immune activation. The resulting inflammatory response may increase blood–brain barrier permeability, activate microglia and astrocytes, promote oxidative stress, and increase susceptibility to postoperative cognitive decline. Solid black arrows indicate relationships supported by mechanistic or experimental evidence, whereas the orange dashed arrow denotes the proposed clinical link to postoperative cognitive dysfunction, for which direct causal evidence remains limited. BBB, blood–brain barrier; DAMPs, damage-associated molecular patterns; FFAs, free fatty acids; HMGB1, high-mobility group box 1; POCD, postoperative cognitive dysfunction; Tregs, regulatory T cells.

## 2 Obesity-induced adipose tissue dysfunction and immune remodeling

### 2.1 Structural and functional remodeling of adipose tissue

Adipose tissue is a metabolically and immunologically active organ that regulates energy storage, endocrine signaling, and systemic homeostasis (20, 21). In obesity, particularly within white adipose tissue, sustained energy excess may preferentially drive adipocyte hypertrophy when adipogenic hyperplasia is insufficient (22, 23). Enlarged adipocytes subsequently develop cellular stress, including endoplasmic reticulum stress, mitochondrial dysfunction, extracellular matrix remodeling, and impaired lipid handling (22–24). As adipose tissue continues to expand, adipocyte death increases and macrophages accumulate around damaged or dying adipocytes, forming characteristic crown-like structures (25–27). These structural alterations represent an early link between adipose tissue expansion and the development of chronic local inflammation.

Adipose tissue expansion also exceeds the capacity of its vascular supply, resulting in tissue hypoxia, dysregulated angiogenesis, and extracellular matrix remodeling (25, 28, 29). Hypoxia promotes adipocyte stress and inflammatory signaling, whereas progressive fibrosis reduces tissue flexibility and limits the capacity of adipose tissue to accommodate excess lipids safely (22, 30). Consequently, surplus lipids may be redistributed to non-adipose organs, contributing to ectopic lipid deposition, lipotoxicity, and systemic metabolic dysfunction (23). The combination of adipocyte hypertrophy, hypoxia, fibrosis, and impaired angiogenesis therefore transforms adipose tissue from an adaptive energy-storage organ into a dysfunctional and chronically stressed tissue (22, 30).

These structural abnormalities are accompanied by impaired endocrine and immunological functions. The secretory profile of obese adipose tissue shifts away from anti-inflammatory and insulin-sensitizing mediators, such as adiponectin, toward increased production of pro-inflammatory adipokines, cytokines, chemokines, and lipid-derived signals (24, 31, 32). Physical inactivity, excessive intake of saturated fats, genetic susceptibility, low birth weight, and sympathetic nervous system overactivity may further promote adipose tissue dysfunction (33). Together, these changes establish a local microenvironment that favors immune-cell recruitment and activation, providing the basis for extensive immune remodeling within obese adipose tissue.

### 2.2 Macrophage accumulation, metabolic reprogramming, and trained immunity

Macrophages are central components of obesity-induced adipose tissue inflammation. Adipocyte hypertrophy, hypoxia, cellular stress, and adipocyte death increase the release of damage-associated molecular patterns and chemotactic mediators (34). In particular, C-C motif chemokine ligand 2, also known as monocyte chemoattractant protein-1 (CCL2/MCP-1), promotes circulating monocyte recruitment through C-C chemokine receptor 2 (35). Recruited monocytes differentiate into adipose tissue macrophages and accumulate around damaged adipocytes, contributing to crown-like structure formation and sustained local inflammation (27, 35, 36).

Obesity also alters the functional phenotype of adipose tissue macrophages. This process has traditionally been described as a shift from an anti-inflammatory M2 phenotype toward a pro-inflammatory M1 phenotype, accompanied by increased production of tumor necrosis factor-alpha (TNF-α), interleukin-1 beta (IL-1β), and interleukin-6 (IL-6) (37, 38). However, macrophages within obese adipose tissue display substantial phenotypic and functional heterogeneity that cannot be adequately represented by a binary M1/M2 classification (39–41). Distinct macrophage populations may simultaneously participate in lipid handling, clearance of damaged cells, tissue remodeling, and inflammatory amplification. Therefore, adipose tissue macrophage activation should be understood as a dynamic spectrum shaped by local metabolic and inflammatory signals (42).

In addition to phenotypic changes, adipose tissue macrophages undergo metabolic reprogramming. Obesity-associated macrophage activation is characterized by increased glycolytic activity, altered mitochondrial metabolism, oxidative stress, and sustained hypoxia-inducible factor 1-alpha (HIF-1α)-dependent inflammatory signaling (43). These metabolic adaptations can support prolonged cytokine production and reinforce adipose tissue inflammation. Chronic exposure to fatty acids, hyperglycemia, and inflammatory mediators may also induce persistent epigenetic and metabolic changes resembling trained immunity, in which innate immune cells develop an exaggerated response to subsequent inflammatory stimulation (44, 45). Although this concept provides a plausible explanation for heightened inflammatory responsiveness in obesity, evidence specifically demonstrating trained immunity in human adipose macrophages remains incomplete, and its perioperative relevance requires direct investigation. A recent review further proposed that obesity can induce maladaptive trained immunity in adipose tissue macrophages, thereby sustaining local inflammation and propagating immunometabolic dysfunction to distant organs. However, its contribution to perioperative inflammatory amplification remains untested (46).

### 2.3 T-cell imbalance and adaptive immune dysregulation

Adaptive immune cells are also important regulators of adipose tissue homeostasis. In lean adipose tissue, regulatory T cells (Tregs) contribute to immune tolerance and restrain excessive inflammatory activation (47). Obesity disrupts this protective environment, leading to increased accumulation and activation of pro-inflammatory CD8^+^ T cells, T helper 1 (Th1) cells, and T helper 17 (Th17) cells, together with reduced abundance or impaired function of adipose-resident Tregs (48). This shift promotes a more inflammatory adaptive immune profile within adipose tissue.

Activated CD8^+^ T cells may precede and facilitate macrophage recruitment, whereas Th1 cells produce interferon-gamma (IFN-γ) and TNF-α, which further enhance macrophage inflammatory activation and impair insulin signaling (49, 50). Th17 cells and IL-17-associated signaling may promote chemokine production and additional immune-cell recruitment (51). Conversely, loss of Treg-mediated immune regulation weakens anti-inflammatory control and permits sustained activation of macrophages and other immune-cell populations. These reciprocal interactions create a local amplification circuit between innate and adaptive immunity (52).

The relative contribution of individual T-cell subsets varies according to adipose depot, obesity duration, metabolic phenotype, and disease stage. Moreover, much of the mechanistic evidence concerning adipose T-cell dysregulation has been derived from experimental models. Although human studies support obesity-associated changes in adipose immune-cell composition, their functional consequences and relationship to systemic inflammatory responses require further clarification. Nevertheless, the imbalance between pro-inflammatory T-cell subsets and Tregs represents an important component of obesity-associated adipose immune remodeling (52).

### 2.4 NLRP3 Inflammasome and TLR4/NF-κB signaling

The NLR family pyrin domain containing 3 (NLRP3) inflammasome functions as an immunometabolic sensor that links cellular stress to inflammatory cytokine maturation. Obesity-associated stimuli, including saturated fatty acids, ceramide accumulation, mitochondrial dysfunction, excessive reactive oxygen species production, and damage-associated molecular patterns, can provide priming or activation signals for the NLRP3 inflammasome, particularly in adipose tissue macrophages (53, 54). Following inflammasome assembly, NLRP3 activates caspase-1, which cleaves pro-IL-1β and pro-IL-18 into their biologically active forms. IL-1β and context-dependent IL-18 signaling subsequently promote immune-cell activation, adipose tissue inflammation, and impaired insulin signaling (55).

Toll-like receptor 4 (TLR4) signaling represents another important interface between lipid stress and innate immune activation. In dysfunctional adipose tissue, saturated fatty acids, damage-associated molecular patterns, and associated endogenous cofactors can promote or potentiate TLR4 signaling in adipocytes and macrophages. Some lipid-associated effects may occur indirectly through molecules such as fetuin-A rather than through direct binding of fatty acids to TLR4 (56, 57). Predominantly through myeloid differentiation primary response 88 (MyD88)-dependent signaling, TLR4 activates nuclear factor kappa B (NF-κB), thereby increasing the expression of TNF-α, IL-6, CCL2/MCP-1, and other inflammatory mediators (58).

NLRP3 and TLR4/NF-κB signaling are closely interconnected. TLR4-mediated NF-κB activation can provide transcriptional priming for NLRP3 and pro-IL-1β, whereas mitochondrial stress and lipid-derived signals facilitate inflammasome activation (59). Persistent activation of these pathways promotes macrophage recruitment, inflammatory cytokine production, and insulin resistance, reinforcing the interaction between immune dysfunction and metabolic stress (53, 59). Current evidence therefore supports their established roles in obesity-associated adipose inflammation (53). However, whether activation of these pathways specifically within adipose tissue directly determines perioperative neuroinflammatory or cognitive outcomes has not been established and should be regarded as a proposed mechanistic connection requiring further validation.

Collectively, obesity induces coordinated structural, metabolic, and immune remodeling within adipose tissue. Adipocyte hypertrophy, hypoxia, fibrosis, macrophage accumulation, T-cell imbalance, and activation of innate immune pathways transform adipose tissue into a persistent source of inflammatory and metabolic signals (22, 53). The circulating consequences of this dysfunctional adipose microenvironment, including altered adipokines, cytokines, chemokines, free fatty acids, and lipid mediators, are discussed in the following section.

## 3 Circulating immunometabolic mediators and systemic metabolic dysfunction

Dysfunctional adipose tissue releases adipokines, cytokines, chemokines, free fatty acids, and bioactive lipid mediators into the circulation, thereby linking local adipose inflammation with systemic immune and metabolic dysfunction (60–64). These mediators interact rather than act independently: inflammation promotes insulin resistance and abnormal lipid handling, whereas metabolic stress further reinforces immune activation. This circulating immunometabolic environment may increase vulnerability to subsequent perioperative stress, although its direct contribution to POCD remains insufficiently established.

### 3.1 Adipokines, cytokines, and chemokines

Obesity substantially alters the adipokine secretory profile of adipose tissue. Circulating leptin concentrations generally increase with adiposity, whereas leptin resistance may limit its metabolic effects (65). In contrast, adiponectin concentrations commonly decline, reducing anti-inflammatory and insulin-sensitizing activity. Resistin may also contribute to systemic inflammation and metabolic dysfunction, although its cellular sources and biological effects differ between humans and experimental models (60, 64). Beyond their metabolic roles, adipokines can regulate immune-cell activity; for example, leptin can activate circulating monocytes and stimulate TNF-α and IL-6 production (66).

Dysfunctional adipose tissue and infiltrating immune cells also release pro-inflammatory cytokines and chemokines. TNF-α interferes with insulin signaling, IL-6 contributes to systemic low-grade inflammation, and inflammasome-dependent IL-1β can amplify innate immune responses (67–71). Meanwhile, CCL2/MCP-1 promotes continued monocyte recruitment into adipose tissue, sustaining macrophage accumulation and inflammatory signaling (36, 63). Together, these mediators extend obesity-associated immune activation beyond adipose tissue.

However, direct perioperative evidence remains limited. Associations between leptin, adiponectin, resistin, and cognitive impairment have mainly been reported outside the surgical setting (72). Moreover, a recent prospective cohort study found no consistent association between preoperative leptin, soluble leptin receptor, or adiponectin concentrations and perioperative neurocognitive disorders (73). Adipokine and cytokine dysregulation should therefore be regarded as a biologically plausible component of the adipose–immune–brain axis rather than an established cause or validated biomarker of obesity-associated POCD.

### 3.2 Free fatty acids and lipid mediators

Impaired lipid storage in dysfunctional adipose tissue increases the release of free fatty acids (FFAs). When the lipid-buffering capacity of adipose tissue is exceeded, excess FFAs may promote ectopic lipid deposition, mitochondrial stress, impaired insulin signaling, and lipotoxicity (74, 75). However, their inflammatory effects depend on fatty acid composition, concentration, exposure duration, and biological context; circulating FFAs should therefore not be regarded as uniformly pro-inflammatory (76).

Polyunsaturated fatty acids are also precursors of bioactive lipid mediators. Arachidonic acid-derived mediators frequently participate in inflammatory initiation, whereas EPA- and DHA-derived mediators may exert anti-inflammatory or pro-resolving effects (77). Dietary n-3 polyunsaturated fatty acids may shift circulating eicosanoid profiles toward a less inflammatory pattern in individuals with overweight or obesity, although clinical findings remain heterogeneous (78). Specialized pro-resolving mediators (SPMs), including lipoxins, resolvins, protectins, and maresins, actively promote the termination of inflammation and restoration of tissue homeostasis. Altered SPM profiles in severe obesity and after bariatric surgery suggest that obesity may involve both persistent inflammation and insufficient resolution responses (79).

Other lipid classes, including ceramides, sphingosine-1-phosphate, and oxidized phospholipids, can influence insulin signaling, immune-cell activity, and vascular function (80, 81). Lipoxins and resolvins have also been investigated in experimental neurological injury, but their relevance to postoperative neuroinflammation remains uncertain (82). Thus, lipid mediators may either amplify inflammation or promote its resolution, but their roles as biomarkers or mechanistic intermediates in obesity-associated POCD require direct investigation.

### 3.3 Insulin resistance, hyperglycemia, and dyslipidemia

Inflammation and metabolic dysfunction form a self-reinforcing cycle in obesity. Pro-inflammatory cytokines impair insulin signaling in adipose tissue, liver, and skeletal muscle, whereas insulin resistance increases adipose lipolysis, circulating FFAs, and ectopic lipid accumulation. Systemic insulin resistance may also be accompanied by impaired brain insulin signaling, altered glucose utilization, mitochondrial dysfunction, and reduced synaptic plasticity (83, 84). However, most supporting evidence originates from studies of diabetes, aging, and neurodegenerative disease, whereas direct evidence from perioperative settings remains limited (85).

Hyperglycemia can further amplify oxidative stress, mitochondrial dysfunction, advanced glycation-related signaling, and endothelial activation. Experimental evidence indicates that high-glucose conditions enhance microglial inflammatory responses through TLR4-associated signaling (86). Nevertheless, acute perioperative hyperglycemia should be distinguished from chronic poor glycemic control, typically reflected by glycated hemoglobin (HbA1c), and their associations with specific postoperative neurocognitive outcomes are considered separately in the clinical evidence section (87).

Dyslipidemia may contribute to endothelial dysfunction, oxidative stress, and abnormal lipid delivery to the brain. Non-perioperative studies have linked altered lipid profiles with impaired blood–brain barrier integrity, whereas high-fat diet models demonstrate reduced hippocampal neurogenesis, altered brain-derived neurotrophic factor signaling, and synaptic dysfunction (88, 89). These findings provide mechanistic plausibility but do not establish a direct causal pathway in surgical patients. Overall, these interacting abnormalities may establish a state of systemic immunometabolic vulnerability that could amplify the inflammatory response to surgical injury, consistent with a proposed “second-hit” framework.

## 4 Perioperative stress as a second inflammatory hit

Obesity-induced adipose immune remodeling may establish a chronically primed immunometabolic state before surgery. This state is characterized by persistent low-grade inflammation, macrophage and T-cell dysregulation, altered adipokine secretion, and impaired metabolic homeostasis (52). Surgical trauma may then act as a second inflammatory hit, superimposing an acute immune response on this pre-existing condition. Tissue injury, neuroendocrine stress, and perioperative metabolic disturbances can trigger a rapid increase in circulating inflammatory mediators, potentially producing a stronger or more prolonged response in patients with obesity (52, 90).

Surgical injury promotes the release of damage-associated molecular patterns, including high-mobility group box 1 (HMGB1), from damaged tissues. HMGB1 can activate innate immune signaling through receptors such as TLR4 and the receptor for advanced glycation end products, thereby stimulating circulating monocytes and macrophages (91, 92). This response is accompanied by increased production of TNF-α, IL-1β, IL-6, and other inflammatory mediators. Experimental studies indicate that TNF-α can initiate a broader cytokine cascade after surgery, whereas IL-1β contributes to surgery-induced inflammatory responses and cognitive impairment (93, 94). These findings support a mechanistic link between acute surgical inflammation and subsequent neuroimmune dysfunction, although most direct evidence is preclinical.

Obesity-associated innate immune memory may further intensify this response. Chronic exposure to a Western diet, weight cycling, fatty acids, and other metabolic stimuli can induce persistent metabolic and epigenetic reprogramming of innate immune cells, resulting in exaggerated responses to later challenges (44, 45). Adipose tissue macrophages and circulating monocytes in this primed state may therefore respond more strongly to surgery-derived inflammatory signals (95). However, whether adipose macrophage trained immunity directly amplifies perioperative inflammation in humans has not been established and remains a proposed mechanism.

Circulating monocytes and macrophages may serve as an important connection between tissue injury and systemic inflammatory amplification. Preclinical evidence indicates that surgery-derived HMGB1 can activate bone marrow-derived monocytes and macrophages, which subsequently contribute to postoperative inflammatory responses (92). At the same time, obesity-associated activation of c-Jun N-terminal kinase, NF-κB, NLRP3, and related pathways may lower the threshold for cytokine production and prolong the resolution of inflammation (45, 96–98). The resulting systemic response may be shaped not only by obesity but also by age, diabetes, frailty, nutritional status, surgical severity, and perioperative complications (90).

The second-hit model therefore provides a plausible framework in which obesity-related immunometabolic dysfunction increases susceptibility to acute surgical inflammation. Nevertheless, direct clinical evidence that patients with obesity develop a disproportionately amplified perioperative inflammatory response remains limited. Although perioperative increases in inflammatory markers have been observed, these findings neither identify adipose tissue as their source nor establish a causal relationship with POCD (99). Because studies of CRP and IL-6 have assessed different neurocognitive outcomes, their associations with POD and POCD are evaluated separately in Section 5. The amplified peripheral inflammatory response may subsequently affect the brain through BBB and neuroimmune pathways.

## 5 Blood–brain barrier disruption and neuroimmune dysfunction

The blood–brain barrier (BBB) is a key interface through which peripheral immunometabolic disturbances may influence the brain. Obesity-associated inflammation, oxidative stress, insulin resistance, and vascular dysfunction can impair BBB integrity (100). During the perioperative period, acute inflammation caused by surgical injury may further activate brain endothelial signaling and disrupt tight-junction function, thereby increasing BBB permeability and facilitating peripheral-to-central inflammatory communication (101–103). Experimental evidence also indicates that IL-17A contributes to perioperative neurocognitive impairment through BBB disruption in aged mice, although its adipose origin and clinical relevance remain unconfirmed (102). Recent evidence further emphasizes that adipose-derived cytokines, adipokines, lipid mediators, and extracellular vesicles may influence the brain through endothelial, BBB, and glial pathways, although most evidence remains non-perioperative (104).

BBB dysfunction can promote microglial and astrocytic activation. Activated microglia release cytokines and reactive oxygen species, whereas reactive astrocytes may further amplify inflammatory signaling and impair neuronal support and BBB homeostasis (1, 71). Experimental studies have demonstrated an important role for microglia in postoperative hippocampal inflammation and cognitive decline (103, 105). Interactions among activated endothelial cells, microglia, and astrocytes may therefore establish a self-amplifying neuroinflammatory response after surgery.

Persistent neuroinflammation can impair neuronal metabolism, mitochondrial function, synaptic plasticity, and neurogenesis. Experimental and non-perioperative studies link obesity-related metabolic inflammation with mitochondrial dysfunction, oxidative stress, abnormal neuronal lipid handling, and reduced hippocampal neurogenesis (89, 106–109). These changes provide a plausible biological basis for increased cognitive vulnerability but do not establish that adipose inflammation directly causes POCD in humans.

Overall, current evidence supports separate associations between obesity and BBB dysfunction and between surgical stress and postoperative neuroinflammation. However, the integrated pathway in which obesity-related adipose inflammation directly amplifies surgery-induced BBB disruption, glial activation, and cognitive decline remains largely inferential. Existing clinical studies do not identify dysfunctional adipose tissue as the source of perioperative neuroinflammatory abnormalities (110). The proposed interactions among adipose tissue dysfunction, circulating immunometabolic mediators, perioperative inflammatory amplification, BBB disruption, and glial–neuronal injury are summarized in **Figure 2**. Recent translational evidence has begun to support this integrated pathway. Tropis et al. reported that obesity aggravated postoperative cognitive changes in patients and experimental animals, while interventions targeting adiposity or inflammation attenuated BBB dysfunction and cognitive impairment in mice (111). Nevertheless, the human findings remain observational and require confirmation in larger cohorts.

**Figure 2:**
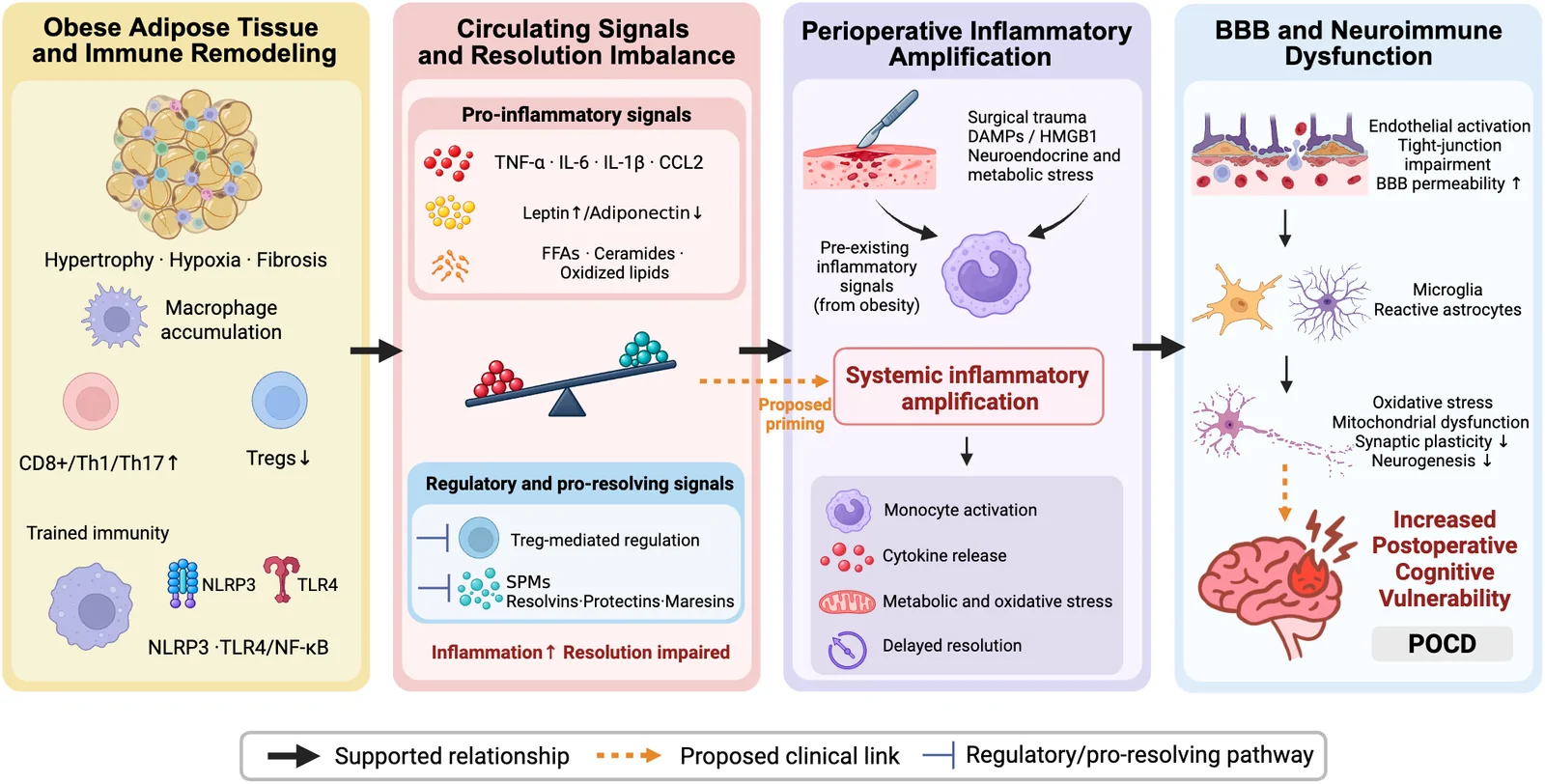
Immunometabolic pathways in the proposed adipose–immune–brain axis underlying postoperative cognitive vulnerability. Obesity induces structural and immune remodeling of adipose tissue, characterized by adipocyte hypertrophy, hypoxia, fibrosis, macrophage accumulation and trained immunity, expansion of pro-inflammatory CD8^+^, Th1, and Th17 cells, reduction of regulatory T cells, and activation of NLRP3 and TLR4/NF-κB signaling. This remodeling promotes the release of pro-inflammatory cytokines, adipokines, free fatty acids, ceramides, and oxidized lipids, while regulatory and pro-resolving mechanisms involving Tregs and specialized pro-resolving mediators may be impaired. Surgical trauma, the release of DAMPs, and neuroendocrine and metabolic stress may amplify this pre-existing inflammatory state, contributing to monocyte activation, cytokine release, metabolic and oxidative stress, and delayed inflammatory resolution. These systemic responses may promote endothelial activation, tight-junction impairment, increased blood–brain barrier permeability, microglial and astrocytic activation, mitochondrial dysfunction, impaired synaptic plasticity and neurogenesis, and ultimately increased postoperative cognitive vulnerability. Solid black arrows indicate supported mechanistic relationships, orange dashed arrows indicate proposed clinical or priming links, and blue inhibitory symbols indicate regulatory or pro-resolving pathways. The overall framework is biologically plausible, but direct clinical evidence establishing adipose inflammation as a causal driver of POCD remains limited. BBB, blood–brain barrier; CCL2, C-C motif chemokine ligand 2; DAMPs, damage-associated molecular patterns; FFAs, free fatty acids; HMGB1, high-mobility group box 1; IL, interleukin; NF-κB, nuclear factor kappa B; NLRP3, NLR family pyrin domain containing 3; POCD, postoperative cognitive dysfunction; SPMs, specialized pro-resolving mediators; TLR4, toll-like receptor 4; TNF-α, tumor necrosis factor-α; Tregs, regulatory T cells.

## 6 Clinical evidence linking obesity-related factors to perioperative neurocognitive outcomes

### 6.1 Obesity and metabolic syndrome

Clinical evidence directly linking obesity to postoperative cognitive dysfunction remains limited and heterogeneous. Although obesity is associated with chronic inflammation, insulin resistance, vascular dysfunction, and cognitive impairment outside the perioperative setting, current studies do not establish obesity itself as an independent risk factor for POCD (15–17). One cohort study found that diabetes, rather than obesity alone, was associated with postoperative cognitive decline, suggesting that metabolic phenotype may be more informative than body mass index (BMI) alone (112). Metabolic syndrome has also been examined in relation to both postoperative delirium (POD) and POCD, but these clinically distinct outcomes should not be interpreted interchangeably (7).

The association between obesity and postoperative cognition is further complicated by age, diabetes, cardiovascular disease, frailty, and malnutrition (17–19). BMI does not distinguish visceral from subcutaneous adiposity or identify differences in insulin resistance, inflammatory burden, and adipose tissue dysfunction. Consequently, patients with a similar BMI may have substantially different perioperative risks. Future clinical studies should characterize obesity using metabolic and inflammatory phenotypes rather than BMI alone (113).

A recent review similarly proposed that metabolic syndrome may prime the perioperative brain through chronic systemic inflammation, BBB vulnerability, central insulin resistance, and mitochondrial dysfunction. This framework supports the importance of metabolic phenotype but does not isolate adipose tissue inflammation as an independent causal factor (114). More recently, Tropis et al. identified obesity as an independent predictor of early postoperative cognitive decline in a mixed cardiac and orthopedic surgical cohort (111). Although this study provides important translational support, its sample size, mixed surgical population, and early assessment period limit extrapolation to longer-term POCD.

### 6.2 Inflammatory and metabolic biomarkers

Inflammatory biomarkers have been investigated in both POD and POCD, but the corresponding evidence must be distinguished. In studies focusing on acute POD, preoperative or early postoperative inflammatory markers, including CRP and IL-6, have been evaluated as potential predictors, although the findings remain variable (115, 116). By contrast, studies of POCD have reported associations between preoperative high-sensitivity CRP and postoperative cognitive impairment after cardiac surgery, as well as associations between postoperative increases in CRP or IL-6 and early or intermediate cognitive decline (117–120). Because POD is an acute fluctuating disturbance of attention and awareness, whereas POCD is identified through decline on neuropsychological testing, biomarker findings from POD studies should not be directly extrapolated to POCD (121).

S100 calcium-binding protein B (S100B) has also been investigated as a marker of astrocytic injury or altered BBB integrity, but its specificity for POCD is limited (110, 122). Evidence for adipokines is less consistent. In a prospective cohort of older surgical patients, preoperative leptin, soluble leptin receptor, and adiponectin concentrations showed no consistent overall associations with either POD or POCD, although exploratory findings suggested possible variation according to obesity status (73). These findings do not support the current use of adipokines as established perioperative cognitive biomarkers.

Metabolic abnormalities may also contribute to postoperative cognitive vulnerability. Perioperative hyperglycemia and diabetes have been associated with adverse neurocognitive outcomes, whereas the influence of obesity alone appears less consistent (112, 123). Lipid-related evidence also differs by outcome: a recent meta-analysis associated hyperlipidemia with acute POD, while a prospective cohort linked hyperlipidemia and total cholesterol with POCD assessed from 7 days to 3 months after surgery (124, 125). A small serum metabolomics study identified a phosphatidylserine species as a potential marker of early POCD, but its sample size limits generalizability (126). These observations support further study of metabolic biomarkers but do not establish causal relationships.

**Table 1** summarizes representative clinical studies and evidence syntheses examining obesity-related metabolic factors, adipokines, lipid abnormalities, and perioperative neurocognitive outcomes. To avoid inappropriate comparison, the studies are grouped according to whether they primarily assessed POD, postoperative cognitive decline or POCD, or mixed and heterogeneous perioperative neurocognitive outcomes.

**Table 1:** Representative clinical studies and evidence syntheses examining obesity-related factors and distinct perioperative neurocognitive outcomes.

Study	Design and population	Metabolic exposure	Neurocognitive outcome and timing	Main finding and limitation
A. Studies primarily assessing postoperative delirium				
Qiu et al., 2025 (124)	Systematic review and meta-analysis of 9 studies involving 4,686 surgical patients	Hyperlipidemia and lipid profiles	POD during the early postoperative period	Hyperlipidemia was associated with increased POD risk. Heterogeneity in surgical populations, lipid definitions, and delirium assessments limits generalizability, and the findings should not be extrapolated directly to POCD.
B. Studies primarily assessing postoperative cognitive decline or POCD				
Qian and Wang, 2020 (126)	Prospective serum metabolomics study of 20 older patients undergoing elective surgery	Metabolomic and lipidomic profiles, including PS(17:2/0:0)	Early POCD assessed using the ISPOCD battery within 7 postoperative days	Several lipid metabolites showed potential diagnostic value for early POCD. The small sample and exploratory design preclude causal interpretation.
Das et al., 2016 (127)	Prospective observational study of 60 patients undergoing off-pump coronary artery bypass surgery	Preoperative statin therapy	Early postoperative memory impairment assessed using a neuropsychological test battery	Statin use was associated with less early memory impairment. The nonrandomized design and small sample introduce potential selection bias.
Tropis et al., 2026 (111)	Prospective single-center study of 106 adults aged ≥60 years undergoing cardiac or orthopedic surgery	Obesity, circulating inflammatory markers, and subcutaneous adipose inflammatory profiles	Early PND assessed on postoperative day 5 using the MMSE or TICS; POD assessed separately for 7 days	Obesity was independently associated with early PND. The single-center design, mixed surgical populations, different cognitive instruments, and short follow-up limit generalizability.
Shen et al., 2025 (125)	Prospective cohort study of 98 patients undergoing laparoscopic gynecological tumor surgery	Hyperlipidemia and total cholesterol	POCD assessed at 7 days, 1 month, and 3 months using the MoCA and TICS-m	Hyperlipidemia was associated with a higher incidence of POCD, and total cholesterol was identified as a possible independent predictor. The modest sample and single-center design are important limitations.
C. Studies assessing mixed or heterogeneous perioperative neurocognitive outcomes				
Feinkohl et al., 2024 (73)	Prospective BioCog cohort of 768 adults aged ≥65 years undergoing elective surgery	Leptin, soluble leptin receptor, free leptin index, and total and high-molecular-weight adiponectin	POD assessed until postoperative day 7 or discharge; POCD assessed at 3 months	No consistent associations were found between adipokine concentrations and POD or POCD. Obesity-stratified findings were exploratory and require independent validation.
Travica et al., 2023 (17)	Umbrella review of meta-analyses of observational studies	Glucose regulation, lipid abnormalities, and other metabolic factors	Heterogeneous postoperative cognitive outcomes assessed from the early postoperative period to several months	Metabolic dysfunction may contribute to postoperative cognitive vulnerability, but heterogeneous definitions, assessment tools, and follow-up periods prevented firm conclusions.
Burns et al., 2023 (15)	Narrative review of obesity and postoperative cognitive dysfunction	Obesity and related metabolic and inflammatory factors	Heterogeneous postoperative cognitive outcomes with variable assessment times	The association is biologically plausible, but direct clinical evidence establishing adipose inflammation as a causal driver of POCD remains limited.

ISPOCD, International Study of Postoperative Cognitive Dysfunction; MMSE, Mini-Mental State Examination; MoCA, Montreal Cognitive Assessment; PND, perioperative neurocognitive disorder; POD, postoperative delirium; POCD, postoperative cognitive dysfunction; PS, phosphatidylserine; TICS, Telephone Interview for Cognitive Status; TICS-m, modified Telephone Interview for Cognitive Status.

Outcome terminology is reported according to the original studies. POD is an acute disorder of attention and awareness and is clinically distinct from POCD and other perioperative neurocognitive disorders; these outcomes should not be interpreted interchangeably. Differences in outcome definitions, assessment instruments, and follow-up periods should be considered when comparing studies.

### 6.3 Current limitations of clinical evidence

Interpretation of the current literature is limited by substantial methodological heterogeneity. Studies use different definitions of POD and POCD, cognitive assessment tools, baseline procedures, diagnostic thresholds, and postoperative follow-up periods (121). Some studies assess cognition within days after surgery, whereas others evaluate outcomes after several weeks or months. Small sample sizes and inconsistent adjustment for age, diabetes, frailty, nutritional status, and surgical severity further limit comparisons across studies (116).

Most importantly, few clinical studies simultaneously assess adipose tissue dysfunction, circulating immunometabolic mediators, BBB integrity, and postoperative cognition. Associations between CRP, IL-6, glucose, lipids, or adipokines and neurocognitive outcomes do not demonstrate that these abnormalities originate from adipose tissue (116). The available evidence therefore supports biological plausibility but does not establish a causal adipose–immune–brain pathway in obesity-associated POCD. Prospective studies using standardized neurocognitive definitions, repeated biomarker measurements, and detailed metabolic phenotyping are required to test this proposed pathway (121).

## 7 Therapeutic implications

### 7.1 Weight management and prehabilitation

Weight-loss interventions, including dietary modification, exercise, and bariatric surgery, can improve metabolic status and reduce some obesity-related inflammatory markers. Intentional weight loss has also been associated with modest improvements in selected cognitive domains (128–130). However, these findings were largely obtained outside the perioperative setting, and it remains unknown whether preoperative weight reduction directly reduces POCD risk. Weight management should therefore be considered part of general metabolic optimization rather than an established strategy for POCD prevention (131).

Prehabilitation combining physical activity, dietary optimization, and correction of nutritional deficiencies may improve perioperative resilience. Its timing and intensity should be individualized, particularly in patients with frailty, sarcopenia, or malnutrition, in whom restrictive dietary interventions may further compromise nutritional status (132). Although n-3 polyunsaturated fatty acids can modify some inflammatory and pro-resolving lipid mediators, their effects on adipose inflammation in human obesity remain inconsistent, and their role in preventing POCD is unknown (133, 134).

### 7.2 Metabolic optimization and incretin-based therapies

Perioperative hyperglycemia, insulin resistance, and diabetes are associated with adverse neurocognitive outcomes, supporting careful assessment of metabolic status (112, 123). Nevertheless, the optimal glucose target for POCD prevention has not been established, and intensive glucose lowering may increase the risk of hypoglycemia (87). Future studies should examine glycemic variability, insulin resistance, lipid profiles, and obesity phenotype together rather than using BMI as the sole metabolic measure (113).

Glucagon-like peptide-1 receptor agonists (GLP-1RAs), such as semaglutide, and dual glucose-dependent insulinotropic polypeptide/GLP-1 receptor agonists, such as tirzepatide, provide a potential link between weight reduction, metabolic improvement, and immune modulation (135). GLP-1RAs improve glycemic control, insulin sensitivity, and lipid metabolism and may indirectly reduce adipose inflammation through loss of adiposity (136). Experimental evidence further suggests that tirzepatide can reduce pro-inflammatory adipose tissue macrophage accumulation and cytokine production in obese mice (137). Semaglutide has also been shown to promote resolution of neuroinflammation and modify microglial, endothelial-cell, and pericyte responses in a mouse model of systemic inflammatory challenge (138). However, these findings are preclinical, and direct evidence that GLP-1RAs or dual incretins prevent POCD is currently lacking. Their perioperative neurocognitive effects, optimal timing, and safety require dedicated clinical investigation.

### 7.3 Modulation of inflammation and resolution pathways

Experimental studies implicate TNF-α, IL-1β, TLR4/MyD88/NF-κB signaling, and NLRP3 inflammasome activation in surgery-induced neuroinflammation and cognitive impairment (93, 94, 139, 140). These pathways may represent potential therapeutic targets, particularly in patients with pre-existing metabolic inflammation. Nevertheless, perioperative immune inhibition may interfere with host defense, wound healing, and tissue repair. Current evidence does not support routine cytokine blockade or inflammasome inhibition for POCD prevention (141).

Promoting inflammation resolution may provide a more balanced approach. Specialized pro-resolving mediators, including resolvins, protectins, and maresins, can regulate macrophage phenotype and facilitate resolution of adipose tissue inflammation (142, 143). Restoration of Treg function may also help rebalance pro-inflammatory and regulatory immune responses (52). However, both strategies remain predominantly preclinical, and their perioperative efficacy, optimal delivery, timing, and safety have not been established.

### 7.4 Blood–brain barrier protection and experimental approaches

Preservation of BBB integrity may limit peripheral-to-central inflammatory signaling. Evidence from traumatic brain injury and cerebral ischemia suggests that BBB protection can attenuate neuroinflammation, but these findings represent cross-disease or preclinical extrapolations (144, 145). Adipose-derived stem-cell extracellular vesicles have also shown immunomodulatory and neuroprotective potential in experimental neurological disorders (146). Their active cargo, cellular targets, pharmacokinetics, and long-term safety remain uncertain, and no direct evidence supports their use in obesity-associated POCD.

Additional approaches targeting cerebral lipid handling or the gut–brain axis may be relevant because obesity alters lipid metabolism, microbial metabolites, and systemic inflammation. However, evidence supporting probiotics, microbiota-directed therapies, or specific lipid-targeted treatments for POCD is currently insufficient (147, 148). These approaches should be presented as research priorities rather than clinical recommendations.

### 7.5 Perioperative management

For patients with obesity, perioperative care should prioritize physiological stability, including appropriate anesthetic and analgesic management, avoidance of prolonged hypotension and hypoxemia, and careful control of glucose and temperature (149). No specific anesthetic technique or drug regimen has been proven to prevent obesity-associated POCD (141, 149). Future studies should determine whether treatment effects vary according to visceral adiposity, diabetes status, inflammatory burden, or metabolic phenotype (113).

Clinical trials should use standardized perioperative neurocognitive terminology and distinguish POD, delayed neurocognitive recovery, and longer-term postoperative neurocognitive disorders (121). Multicenter studies should combine neuropsychological testing with repeated measurements of inflammatory, metabolic, and BBB-related biomarkers (121, 141). At present, metabolic optimization, immune modulation, BBB protection, and incretin-based therapies should be considered promising research directions rather than established interventions for POCD prevention (141).

## 8 Limitations and future directions

Current evidence linking obesity-related adipose inflammation to POCD remains predominantly indirect or preclinical (111). Clinical studies vary in neurocognitive definitions, assessment tools, follow-up periods, and adjustment for diabetes, age, frailty, and nutritional status (116, 121). Moreover, few studies directly measure adipose tissue inflammation, and available associations with circulating biomarkers do not establish adipose tissue as their source or confirm causality (111, 116).

Future multicenter prospective studies should use standardized definitions that distinguish POD, delayed neurocognitive recovery, and longer-term postoperative neurocognitive disorders (121). Longitudinal assessment should integrate adipose tissue phenotype, circulating immunometabolic mediators, BBB-related markers, and cognitive outcomes (111, 116). Obesity should also be characterized using visceral adiposity, insulin resistance, and inflammatory phenotype rather than BMI alone (113). Such studies will be essential to validate the proposed adipose–immune–brain pathway and identify clinically relevant therapeutic targets.

## 9 Conclusions

Obesity-induced adipose tissue dysfunction may create a state of chronic immunometabolic vulnerability that is further amplified by surgical stress, potentially promoting BBB disruption, neuroinflammation, and postoperative cognitive decline. Although this adipose–immune–brain axis is biologically plausible, direct clinical evidence remains limited, and a causal relationship between adipose inflammation and POCD has not been established. This framework should therefore guide future mechanistic and prospective clinical studies rather than be interpreted as a confirmed disease pathway.
